# Cutaneous metastasis of esophageal large cell neuroendocrine carcinoma presenting as a solitary cheek nodule with overlapping features of wnt/β-catenin-activated rosette-forming carcinoma

**DOI:** 10.1016/j.jdcr.2026.07.013

**Published:** 2026-07-15

**Authors:** Bryce Demoret, Nick Hess, Jill Browning, Renee M. Thomas

**Affiliations:** a Department of Dermatology, HCA Healthcare/USF Morsani College of Medicine GME: HCA Florida Largo Hospital, Largo, Florida; b Department of Medicine, Division of Dermatology, Ohio University Heritage College of Osteopathic Medicine, Dublin, Ohio; c Department of Dermatology, C.W. Bill Young VA Medical Center, Bay Pines, Florida; d Department of Dermatology and Dermatologic Surgery, Medical University of South Carolina, Charleston, South Carolina

**Keywords:** cutaneous malignancy, cutaneous metastasis, esophageal carcinoma, gastrointestinal malignancy, neuroendocrine carcinoma

## Introduction

Cutaneous metastases from esophageal malignancies are rare and most commonly arise from adenocarcinoma or squamous cell carcinoma of the esophagus.^1^ In contrast, large cell neuroendocrine carcinoma of the esophagus (LCNEC) is an exceptionally uncommon, high-grade neoplasm, accounting for only a minute fraction of all neuroendocrine tumors and typically presents with advanced visceral disease at time of diagnosis.^2^ To our knowledge, cutaneous involvement of esophageal LCNEC has not been documented, making its recognition in the skin particularly challenging. This case describes a diagnostically complex case of esophageal LCNEC that first manifested as a rapidly enlarging cutaneous cheek nodule. The unusual site of metastasis and the tumor’s overlapping basaloid and neuroendocrine features created a tumor classification challenge due to overlapping features with primary cutaneous adnexal and neuroendocrine neoplasms.

## Case presentation

**Fig 1:**
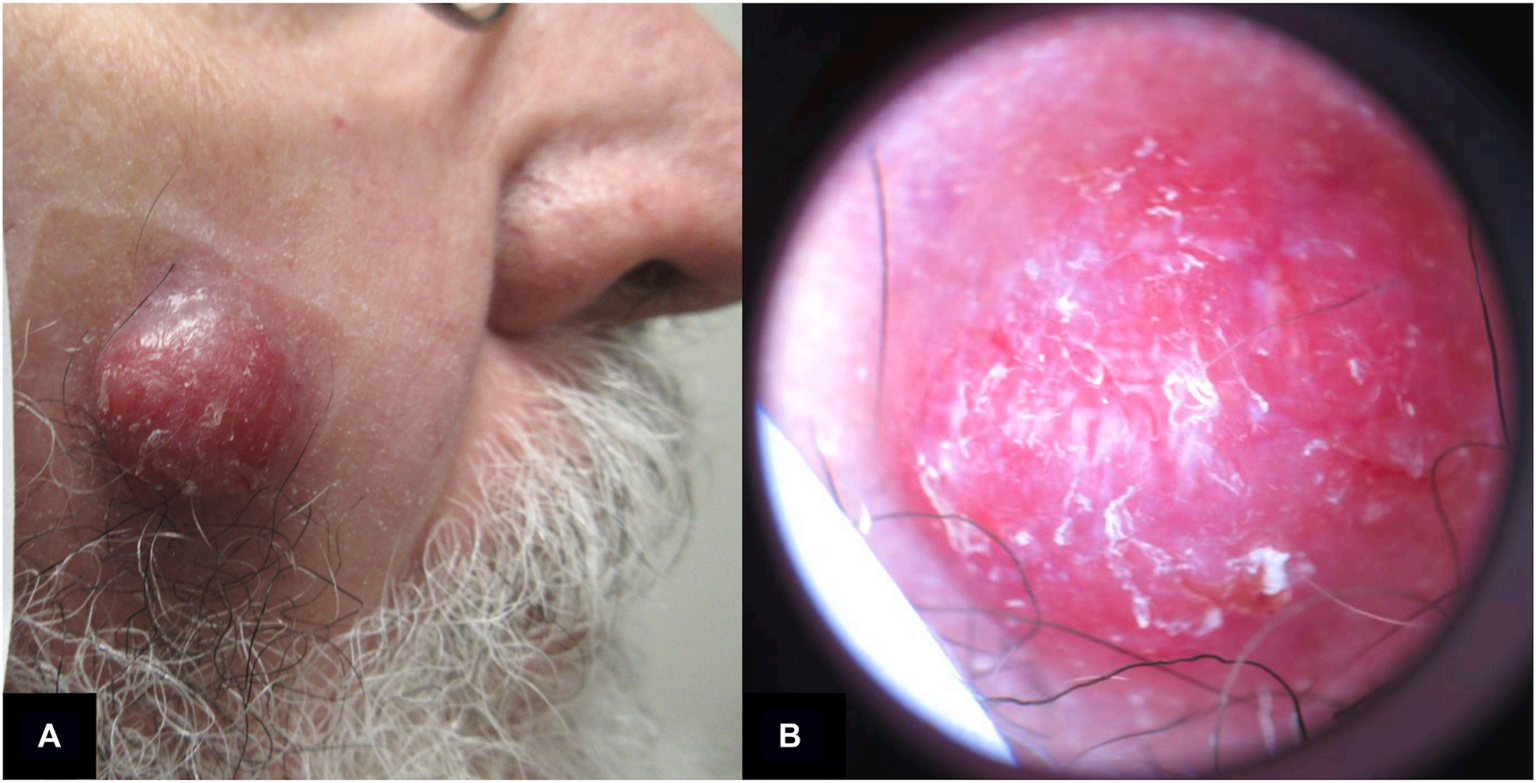
Clinical appearance and dermoscopy. **A,** Solitary 1.7 cm pink-red nodule with minimal superficial scale on the right cheek. **B,** Dermoscopy demonstrated a pink-red nodule with shiny white structures and no discernible pigment network or prominent vascular pattern.

A 79-year-old male with a medical history of hypertension, hyperlipidemia, cervical spine osteoarthritis, and obesity presented to the dermatology clinic for evaluation of a new lesion on the right cheek (Fig 1, *A* and *B*). The patient noted the appearance of the nodule approximately 8 weeks prior to presentation, with rapid growth of the lesion prompting him to seek care. He denied associated pain, pruritus, constitutional symptoms, or prior history of malignancy, but endorsed a 20-30 pack-year history of smoking and chewing tobacco prior to quitting 30 years ago.

**Fig 2:**
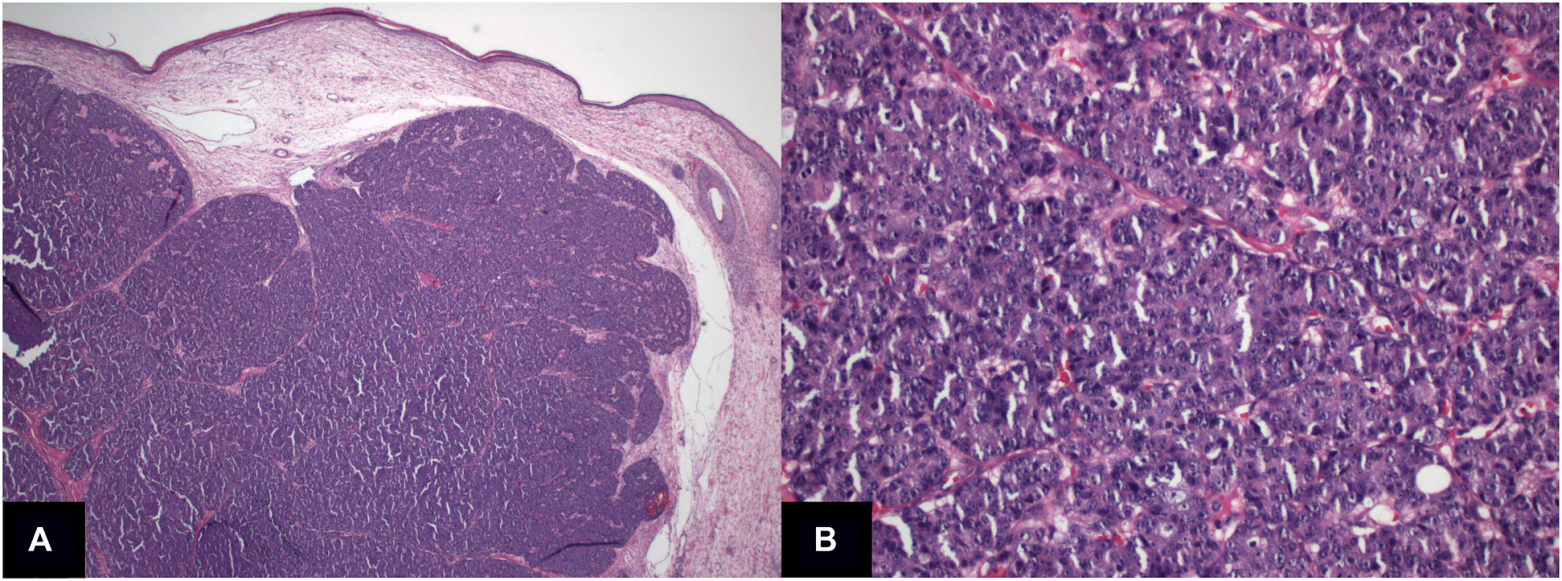
Histologic examination showed: a dermal basaloid neoplasm without epidermal connection **(A)** composed of highly atypical large cells with rosette and pseudoglandular morphology **(B)**; Hematoxylin-eosin staining: 20× and 100× magnification, respectively.

A deep shave biopsy was performed and revealed a dermal-based basaloid neoplasm without connection to the overlying epidermis. The tumor exhibited rosette and pseudoglandular architecture, marked cytologic atypia, and frequent mitotic figures (Fig 2).

**Fig 3:**
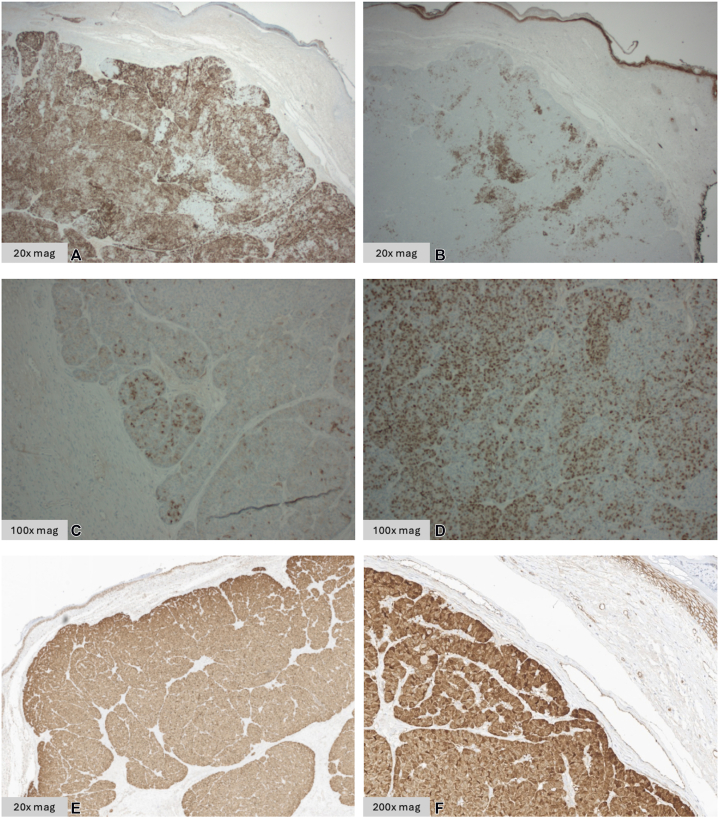
Immunostaining demonstrates strong and diffuse positivity for CD56 **(A)**, positive pankeratin **(B)**, patchy synaptophysin **(C)**, CDX2 **(D)**, and nuclear and cytoplasmic beta-catenin **(E** and **F)**.

Immunohistochemical (IHC) studies highlighted tumor cells that stained positive for pankeratin, CD56, epithelial membrane antigen, Ber-EP4, with patchy positivity for synaptophysin and CDX2 (Fig 3). Tumor cells were negative for CK7, CK20, chromogranin, SOX10, TTF-1, INSM1, p63, and p40. These findings supported a diagnosis of poorly differentiated carcinoma with neuroendocrine features within the dermis. Additional IHC studies showed nuclear and cytoplasmic positivity of β-catenin.

The tumor was submitted for next generation sequencing (NGS). NGS identified a pathogenic ERBB2 mutation, frameshift variants in ARID1A, and low tumor mutational burden (TMB). Taken together with the histopathology and IHC studies, a basaloid adnexal carcinoma with neuroendocrine features favoring a Wnt/β-catenin activated rosette forming carcinoma was considered; however, cutaneous metastasis from underlying carcinoma could not be excluded.

Given concern for possible metastatic disease, a positron emission tomography/computed tomography scan was obtained. Imaging demonstrated an intensely fluorodeoxyglucose-avid mass involving the distal esophagus and gastroesophageal junction, as well as fluorodeoxyglucose-avid right pericardial lymphadenopathy, multiple right lung nodules, and 2 hepatic lesions. No additional suspicious cutaneous lesions were identified. Upper endoscopy revealed a large, partially obstructing malignant tumor in the distal esophagus. Biopsy showed a poorly differentiated carcinoma with neuroendocrine features consistent with large cell neuroendocrine carcinoma. IHC staining of the esophageal tumor was positive for pankeratin, CD56, patchy synaptophysin, patchy CDX2, and negative for CK7, CK20, chromogranin, p40, and p63, mirroring the immunophenotype of the cutaneous lesion.

Given the patient’s preference, age, and presence of widespread metastatic disease at diagnosis, treatment intent was palliative. Systemic immunotherapy with pembrolizumab was considered due to esophageal neuroendocrine tumors commonly expressing PD-1 and PD-L1; however, his tumor markers returned negative rendering him a poor candidate.^3^ The patient underwent percutaneous endoscopic gastrotomy tube placement due to progressive obstruction. Palliative radiation therapy to the esophageal primary lesion and chemotherapy options were also discussed with oncology.

## Discussion

Considering the clinical history, imaging findings, and concordant histopathologic and IHC features, a final diagnosis of esophageal LCNEC presenting as a cutaneous metastasis was established. Cutaneous metastases from esophageal carcinomas are rare and typically occur in the setting of advanced disease.^1^ LCNEC of the esophagus is exceptionally rare, representing less than 0.04% of all neuroendocrine tumors, and while visceral metastases are frequently observed at diagnosis (57%), cutaneous metastasis have not been previously reported.^2^^,^^4^ To our knowledge, this represents the first case of an esophageal LCNEC cutaneous metastasis. Notably, the rapidly growing cutaneous lesion was the patient’s initial presenting symptom and preceded identification of the primary tumor and extensive visceral metastases.

This case underscores several important diagnostic challenges. As highlighted in prior case reports, overlapping IHC profiles can obscure tumor origin and delay diagnosis.^1^^,^^2^ Basaloid cutaneous neoplasms with neuroendocrine differentiation may closely mimic Merkel cell carcinoma, primary adnexal carcinomas, or other rare entities particularly when rosette formation and β-catenin activation are present. CDX2 serves as a key marker of gastrointestinal differentiation and may also be expressed in other tissues, such as skin adnexal tumors with matrical features, through activation of the Wnt/β-catenin pathway.^5^^,^^6^ The histopathological impression of a Wnt/β-catenin–activated rosette-forming carcinoma aligns with a 2024 case series by Kervarrec *et al* describing a unique skin tumor characterized by rosette architecture and evidence of squamous or neuroendocrine differentiation.^6^ Both esophageal LCNEC and the recently defined Wnt/β-catenin–activated rosette-forming carcinoma share overlapping histopathologic and IHC features, including rosette/pseudoglandular architecture, positive IHC staining for epithelial membrane antigen, BerEP4, CDX2, synaptophysin, β-catenin, and IHC negative staining for SOX10, p40, p63, TTF1. However, most reported Wnt/β-catenin–activated rosette-forming carcinoma cases demonstrated an epidermal connection, CK7 positivity (94%), and NGS aberrations in the APC/CTNNB1 pathway, findings absent in our case.^6^ The authors of that series emphasized that metastatic, poorly differentiated gastrointestinal adenocarcinoma should remain an important diagnostic consideration in such presentations. Lastly, TMB may assist in delineating cutaneous from noncutaneous tumor origin. UV signature-positive tumors in large pan-cancer NGS datasets carry a median TMB of ∼31.3 mut/Mb.^7^ The low TMB observed in our case (8.6 mut/Mb) falls well below this threshold, a finding consistent with the absence of significant UV-mediated mutagenesis and lending further genomic support to a noncutaneous primary.

We believe this case represents the first reported instance of cutaneous metastasis arising from esophageal LCNEC. It underscores the importance of maintaining a broad differential diagnosis when evaluating rapidly enlarging cutaneous nodules, even in patients without a prior history of malignancy. Notably, molecular alterations within the Wnt/β-catenin signaling pathway may occur in both primary cutaneous adnexal neoplasms and visceral malignancies, potentially limiting their diagnostic specificity. Comprehensive assessment incorporating IHC profiling, molecular analysis, and systemic imaging remains essential in the evaluation of ambiguous basaloid or neuroendocrine tumors of the skin.

## Conflicts of interest

None disclosed.

## References

[1] Triantafyllou S., Georgia D., Gavriella-Zoi V. (2015). Cutaneous metastases from esophageal adenocarcinoma. Int Surg.

[2] Awada H., Hajj Ali A., Bakhshwin A., Daw H. (2023). High-grade large cell neuroendocrine carcinoma of the esophagus: a case report and review of the literature. J Med Case Rep.

[3] Roberts J.A., Gonzalez R.S., Das S., Berlin J., Shi C. (2017). Expression of PD-1 and PD-L1 in poorly differentiated neuroendocrine carcinomas of the digestive system: a potential target for anti-PD-1/PD-L1 therapy. Hum Pathol.

[4] National Cancer Institute SEER incidence data, 1975–2019: National Cancer Institute: Surveillance, Epidemiology, and End Results Program. https://seer.cancer.gov/data/.

[5] Tekin B., Kundert P., Yang H.H., Guo R. (2022). CDX2 expression in primary skin tumors-case series and review of the literature. Hum Pathol.

[6] Kervarrec T., Cheok Lei K., Sohier P. (2024). Wnt/β-catenin-activated nonpilomatrical carcinoma of the skin: a case series. Mod Pathol.

[7] Mata D.A., Williams E.A., Sokol E. (2022). Prevalence of UV mutational signatures among cutaneous primary tumors. JAMA Netw Open.

